# Surgical technique for removal of high-density silicone oil (Oxane HD)

**DOI:** 10.1186/s40942-023-00501-9

**Published:** 2023-10-23

**Authors:** Ramon Antunes De Oliveira, Nilva Simeren Bueno De Moraes, Rodrigo Antonio Brant Fernandes, Octaviano Magalhães, Mauricio Maia

**Affiliations:** 1 Department of Ophthalmology, Universidade Federal de São Paulo (UNIFESP/EPM), São Paulo, Brazil; 2 Hospital de Olhos Oeste Paulista, Assis, Brazil

**Keywords:** Heavy silicone oil, Vitrectomy, Instrumentation

## Abstract

The main drawback for the use of heavy silicone oil (HSO) Oxane HD is the difficulties it presents during removal. It differs from conventional silicone oil removal because it progressively concentrates in the posterior pole during the removal because of its heavier than water density. We describe a technique to facilitate proper HSO Oxane HD removal and minimize residual posterior bubbles and intraoperative complications. We describe our pearls for removing HSO to obtain the optimal results. A concern associated with HSO is the difficulty it presents when it is removed. However, this can be overcome by appropriate modification of surgical techniques during removal to limit intraoperative complications.

**Supplementary Information:**

The online version contains supplementary material available at 10.1186/s40942-023-00501-9.

## Background

Vitrectomy is the most common surgical technique performed to treat rhegmatogenous retinal detachment, and the most common vitreous substitutes used are air, expandable gases, and silicone oil (SO). Heavy silicone oil (HSO) is an alternative hydrophobic agent and heavier than water that provides a good tamponade effect in the inferior retina. The potential indications for the use of HSO are retinal detachments with large inferior breaks, complicated cases with redetachments and/or inferior proliferative vitreoretinopathy, cases requiring inferior retinotomies, glaucomatous eyes with previously implanted drainage valves located superiorly, and patients incapable of proper head positioning, such as patients with spinal osteoarthritis, or cognitive impairment such as autism or dementia [1]. However, HSO removal can be tricky for inexperienced surgeons because it concentrates in the posterior pole during its removal and requires special attention to mitigate intraoperative complications.

## HSO removal technique

The HSO removal requires a four-port bimanual technique performed under direct visualization. An infusion line is placed followed by positioning of Chandelier illumination. One hand maintains the active aspiration (vacuum, 600 mmHg) and the second hand controls passive drainage using two 18-gauge needles from temporal and nasal sclerotomies. The needle is kept inside the bubble during the entire removal procedure to avoid leaving a residual SO bubble in the posterior pole. The use of the second needle helps mobilize the final HSO bubble into the needle and prevent its release back to the posterior pole. Video Supplemental Digital Content 1 and 2 demonstrates the HSO removal technique in complex retinal detachment surgeries.

Another point to highlight is the importance of placing the sclerotomy removal site as temporal as possible to ease temporal eye tilting and dislocation of the SO bubble outside the macula. Suturing the sclerotomy is mandatory to prevent postoperative leakage.

Use of 20-gauge peripheral intravenous catheters instead of 18-gauge needles is possible. However, we prefer to use the needles because the catheter frequently clogs due to the Oxane HD® (Bausch & Lomb, Toulouse, France) viscosity and usually needs to be replaced. The use of this bimanual technique facilitates the removal of smaller residual SO bubbles that stick to the outside of the cannula and may be scraped off when the cannula is removed from the trocars [2, 3].

The surgery to remove the HSO must be performed carefully to avoid intraoperative complications. For example, high-pressure active aspiration in the posterior pole may damage the retinal and optic disc tissues, possibly resulting in retinal holes [4–6]. Moreover, intraoperative hypotony occurs if an active aspiration cannula is placed in the balanced saline solution rather than into the SO bubble or due to clogging of the infusion line [4]. Eyes with media opacity such as corneal scarring or lens blurring require extra care [7].

Another noteworthy consideration is the tubeless siphon effect of a non-Newtonian fluid. It happens when the SO converts to a tunnel shape during active aspiration and the HSO is aspirated from the posterior pole, avoiding the proximity of the needle to the retina [3, 8].

Other techniques have been described for Densiron® HSO (Fluoron, Gmbh, Neu-Ulm, Germany) removal but they are not appropriate for Oxane HD in our opinion. One is injecting perfluorocarbon liquid (PFCL) posteriorly to the HSO and leaving a 20-gauge sclerotomy open for HSO drainage [4]. This technique has not been accepted widely because previous studies have suggested that the SO that forms becomes sticky when these two compounds are mixed [3]. Although most cases of sticky SO occur when PFCL remnants are in persistent contact with SO or HSO, one case of sticky SO formation was described intraoperatively. It that case, the surgeon exchanged PFCL for SO (fluid-fluid exchange) and removed the SO during the same surgery to retreat residual subretinal fluid [9, 10].

Another procedure is the use of the blue connector from the Constellation Viscous fluid Control Pak® (Alcon, Ft. Worth, TX) connected to a 23-gauge trocar. It reduces Densiron® HSO removal time by combining suction with a short cannula. After the removal, a flute cannula is used to remove residual bubbles in the posterior pole [3]. In our experience, the higher viscosity of Oxane HD (3.300) compared to that of Densiron (1.400) makes this technique difficult because the cannulas usually clog and need to be replaced. This would result in residual posterior bubbles that need to be aspirated in the posterior pole with increased risk to the retina [11].

Active aspiration using a 7.5-mm 20-gauge needle is another technique. According to Poiseuille’s law (flow = ∆Pπr^4^/8Ln), the use of a shorter needle compensates for a smaller diameter and maintains sufficient flow to aspirate HSO Densiron [8]. In our experience, Oxane HD is more viscous and the flow is compromised. In addition, if the tip of the needle looses contact with the HSO, the bubble falls to the posterior pole and cannot be reached using the short cannula. This must be considered, especially in myopic eyes with longer axial lengths. The 18-gauge needle has a greater diameter to override the limitation of the greater length (40 mm) and still achieves sufficient flow to fully aspirate the HSO. In both scenarios, sclerotomies must be sutured to prevent leakage and postoperative hypotony.

The main concern associated with HSO use for most retina surgeons is the difficulty it presents when it is removed. However, appropriate modification of surgical techniques during removal of Oxane HD SO is advised to limit intraoperative complications. These issues notwithstanding, heavy SO remains a useful vitreous substitute in selected high-risk retinal detachment surgery cases.

### Electronic supplementary material

Below is the link to the electronic supplementary material.

Supplementary Material 1

Supplementary Material 2

## Data Availability

Data are available upon reasonable request.

## Abbreviations

HSO: Heavy silicone oil
SO: Silicone oil
PFCL: Perfluorocarbon liquid

## References

[1] Cazabon S, Hillier RJ, Wong D (2011). Heavy silicone oil: a novel intraocular tamponade agent. Optom Vis Sci.

[2] Er H (2010). Primary heavy silicone oil usage in inferior rhegmatogenous retinal detachment. Ophthalmologica.

[3] Romano MR, Groenwald C, Das R, Stappler T, Wong D, Heimann H (2009). Removal of Densiron-68 with a 23-gauge transconjunctival vitrectomy system. Eye (Lond).

[4] Falavarjani KG, Modarres M (2011). Heavy silicone oil removal without a suction pump: a surgical technique. Retina.

[5] Henderson RH, Reddie IC, Campbell WG (2012). A novel technique for high-density silicone oil removal. Retina.

[6] Macías-Murelaga B, Ruiz M, Bascarán L, Gibelalde A, Aldazabal M, Irigoyen C (2013). Aceite de silicona pesado (Densiron(®) 68) en vitreorretinopatía proliferativa: 4 años de experiencia [Heavy silicone oil (Densiron® 68) in proliferative vitreoretinopathy: 4 years of experience]. Arch Soc Esp Oftalmol.

[7] Deobhakta A, Rosen R (2020). Retinal tamponades: current uses and Future Technologies. Curr Ophthalmol Rep.

[8] Stappler T, Williams R, Gibran SK, Liazos E, Wong D (2008). A guide to the removal of heavy silicone oil. Br J Ophthalmol.

[9] Romano MR, Vallejo-Garcia JL, Parmeggiani F, Romano V, Vinciguerra P (2012). Interaction between perfluorcarbon liquid and heavy silicone oil: risk factor for sticky oil formation. Curr Eye Res.

[10] Fukumoto M, Nishida Y, Kida T, Sato T, Kobayashi T, Ikeda T (2018). A case of silicone oil adhered to the retinal surface via perfluorocarbon liquid. BMC Ophthalmol.

[11] Wolf S, Schön V, Meier P, Wiedemann P (2003). Silicone oil-RMN3 mixture (heavy silicone oil) as internal tamponade for complicated retinal detachment. Retina.

